# Titration of C-5 Sterol Desaturase Activity Reveals Its Relationship to Candida albicans Virulence and Antifungal Susceptibility Is Dependent upon Host Immune Status

**DOI:** 10.1128/mbio.00115-22

**Published:** 2022-04-05

**Authors:** Jessica Regan, Christian DeJarnette, Arturo Luna-Tapia, Josie E. Parker, Parker Reitler, Stacey Barnett, Katie M. Tucker, Steven L. Kelly, Glen E. Palmer

**Affiliations:** a Department of Pharmaceutical Sciences, College of Pharmacy, University of Tennessee Health Sciences Center, Memphis, Tennessee, USA; b Department of Molecular Immunology and Biochemistry, College of Graduate Health Sciences, University of Tennessee Health Sciences Center, Memphis, Tennessee, USA; c Department of Clinical Pharmacy and Translational Science, College of Pharmacy, University of Tennessee Health Sciences Center, Memphis, Tennessee, USA; d Institute of Life Science, Swansea University Medical School, Swansea, Wales, United Kingdom; e Laboratory Animal Care Unit, University of Tennessee Health Sciences Center, Memphis, Tennessee, USA; Duke University Medical Center

**Keywords:** antifungal efficacy, C-5 sterol desaturase, *Candida albicans*, ERG3, fluconazole, pathogenicity, virulence, yeasts

## Abstract

The azole antifungals inhibit sterol 14α-demethylase (S14DM), which depletes cellular ergosterol and promotes synthesis of the dysfunctional lipid 14α-methylergosta-8,24(28)-dien-3β,6α-diol, ultimately arresting growth. Mutations that inactivate sterol Δ^5,6^-desaturase (Erg3p), the enzyme that produces the sterol-diol upon S14DM inhibition, enhances Candida albicans growth in the presence of the azoles. However, *erg3* null mutants are sensitive to some physiological stresses and can be less virulent than the wild type. These fitness defects may disfavor the selection of null mutants within patients. The objective of this study was to investigate the relationship between Erg3p activity, C. albicans pathogenicity, and the efficacy of azole therapy. An isogenic panel of strains was constructed that produce various levels of the *ERG3* transcript. Analysis of the sterol composition confirmed a correspondingly wide range of Erg3p activity. Phenotypic analysis revealed that even moderate reductions in Erg3p activity are sufficient to greatly enhance C. albicans growth in the presence of fluconazole *in vitro* without impacting fitness. Moreover, even low levels of Erg3p activity are sufficient to support full virulence of C. albicans in the mouse model of disseminated infection. Finally, while the antifungal efficacy of fluconazole was similar for all strains in immunocompetent mice, there was an inverse correlation between Erg3p activity and the capacity of C. albicans to endure treatment in leukopenic mice. Collectively, these results establish that relative levels of Erg3p activity determine the antifungal efficacy of the azoles upon C. albicans and reveal the critical importance of host immunity in determining the clinical impact of this resistance mechanism.

## INTRODUCTION

Mortality rates associated with invasive fungal infections (IFIs) remain alarmingly high, despite the availability of three major classes of antifungal drugs (1). The azole antifungals block production of the membrane lipid ergosterol through inhibition of sterol 14α-demethylase (S14DM; Erg11p). This leads to depletion of cellular ergosterol and the accumulation of abnormal sterol species, such as 14α-methylergosta-8,24(28)-dien-3β,6α-diol, which compromise membrane function and therefore fungal growth (2). Several genetically encoded mechanisms are known to reduce the sensitivity of Candida albicans, one of the most important human fungal pathogens, to the azole antifungals. This includes mutations that reduce the target enzyme’s affinity for the azoles (3–5) and elevated expression of the target protein or of drug efflux pumps (6, 7). However, a combination of these mechanisms is often necessary to confer clinically relevant levels of resistance. In contrast, mutations that inactivate sterol Δ^5,6^-desaturase (Erg3p), the enzyme responsible for producing the “toxic” diol species upon inhibition of S14DM, leads to the accumulation of 14α-methylfecosterol instead, which is apparently compatible with C. albicans growth (2). However, while azole-resistant *erg3* null mutants can be readily selected *in vitro* (8), they are less commonly reported among azole-resistant clinical isolates (2, 9–11). This could be because loss of Erg3p function itself blocks a late step in ergosterol biosynthesis (10) and, therefore, alters membrane composition and function. As a result, C. albicans *erg3* null mutants are sensitive to some physiological stresses (12–15) and, under some conditions, have reduced hyphal growth (10, 13, 16), a major virulence determinant of this species (17, 18). In addition, some clinically derived and laboratory-produced *erg3* null C. albicans isolates have been reported to be less virulent than the wild type in the standard mouse model of disseminated infection (9, 13, 16, 19). Thus, while complete inactivation of Erg3p leads to azole insensitivity, the associated pathogenicity defects may oppose the selection of null mutants within patients. However, we recently described a C. albicans strain with reduced *ERG3* transcription as insensitive to fluconazole *in vitro* but unaffected in stress tolerance, hyphal growth, or virulence (19). Azole-resistant clinical isolates have also been identified with low levels of sterol Δ^5,6^-desaturase activity (11). This suggests there is a sweet spot, or even a range of Erg3p activity levels, which enable C. albicans to survive and proliferate in the presence of the azoles without significantly compromising its physiological fitness or pathogenicity. However, to date, essentially all attempts to evaluate the importance of *ERG3* as a determinant of azole susceptibility within the mammalian host have sought to identify and characterize null mutants completely lacking sterol Δ^5,6^-desaturase activity. As such, the effects of more subtle differences in Erg3p activity may have been overlooked and the importance of *ERG3* as a determinant of antifungal sensitivity underestimated. The objective of this study was to investigate the relationship between *ERG3* expression levels, azole sensitivity, and the pathogenic fitness of C. albicans.

## RESULTS

### Low levels of *ERG3* transcription are sufficient to support Candida albicans stress tolerance and hyphal growth.

**FIG 1:**
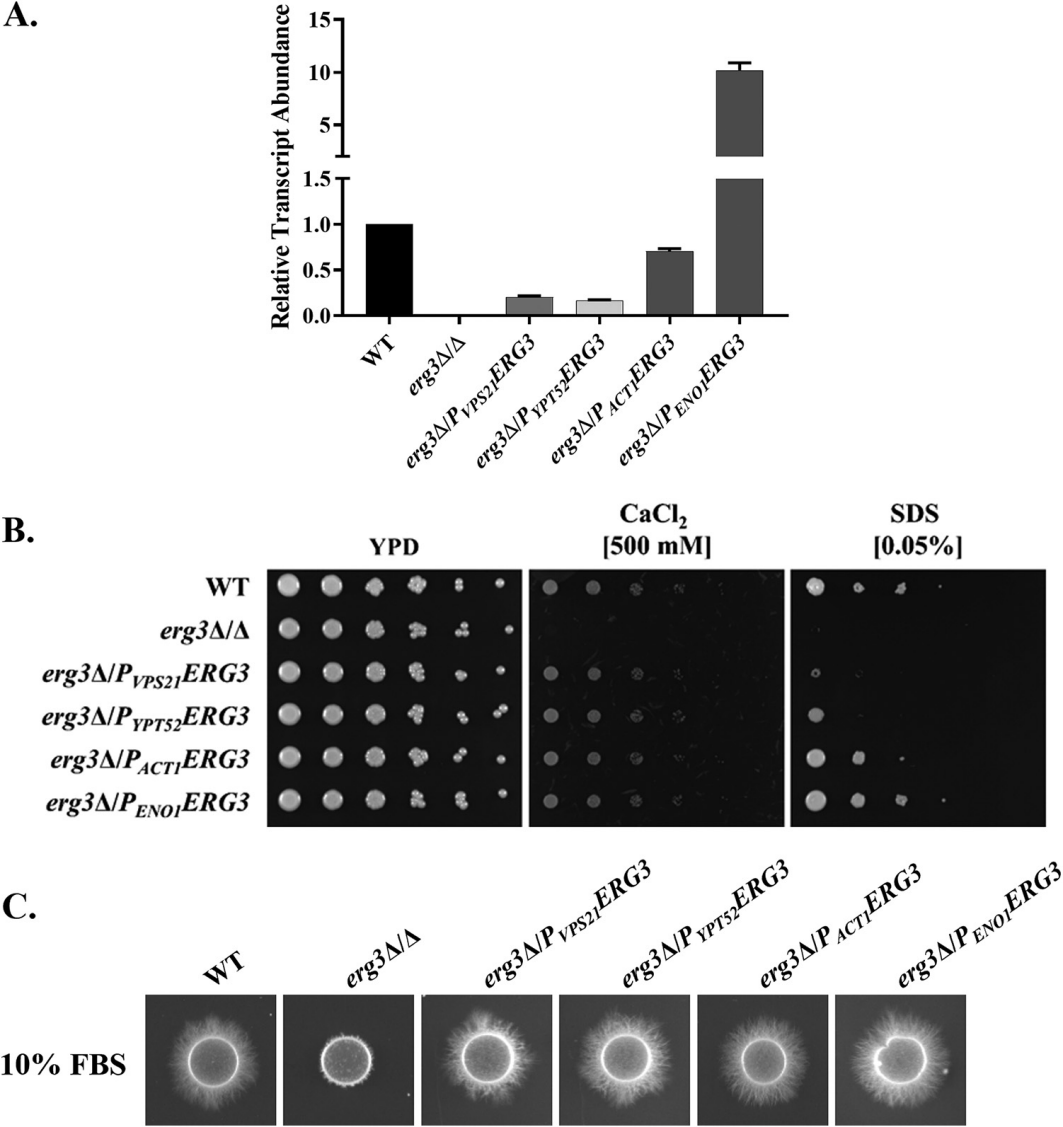
Low levels of *ERG3* transcription are sufficient to support Candida albicans stress tolerance and hyphal growth. (A) C. albicans strains engineered to transcribe the *ERG3* ORF from either the *P_ENO1_*, *P_ACT1_*, *P_YPT52_*, or *P_VPS21_* promoters were grown in YPD medium at 30°C and total RNA extracted. The relative abundance of the *ERG3* transcript was then quantified by qRT-PCR, normalized to levels of the *ACT1* transcript, and expressed relative to the wild-type control. Data are the means from three biological/technical replicates, with error bars indicating the standard deviations. A wild-type (WT; *ERG3*/*ERG3*) strain, SC5314, and the *erg3Δ/Δ* mutant were included for reference. (B) The same strains were suspended at 1 × 10^7^ cells/mL in sterile water and serially diluted 1:5, and each cell suspension was applied to YPD agar or YPD agar supplemented with 500 mM CaCl_2_ or 0.05% SDS. Plates were imaged after 2 days at 30°C. (C) Each strain was suspended at 1 × 10^7^ cells/mL and 2.5 μL spotted onto 10% FBS agar. Colonies were imaged after 4 days at 37°C.

To determine how the level of *ERG3* transcription influences C. albicans fitness and azole susceptibility, we constructed a series of strains in which one allele was deleted and the 5′-untranslated region (UTR) of the second allele was replaced by one of four different C. albicans promoter sequences (20). This produced an isogenic panel of strains with a range of *ERG3* transcript levels and removed any regulatory elements within the native promoter. Quantitative RT-PCR (qRT-PCR) analysis of RNA extracted from these strains confirmed that the relative abundance of the *ERG3* transcript in two of the resulting genotypes, harboring either the *P_VPS21_* or *P_YPT52_* promoters, was approximately 20% that of the wild-type control (Fig. 1A). The *ERG3* transcript was approximately 80% of the wild-type level in the *P_ACT1_-ERG3* strain and ∼10-fold higher than the wild type in the *P_ENO1_-ERG3* strain. As expected, no *ERG3* transcript was detected in the *erg3Δ/Δ* deletion strain. To provide a further measure of C-5–sterol desaturase activity, we compared the sterol composition of each strain by gas chromatography-tandem mass spectrometry (GC-MS/MS) (Table 1). Ergosterol was the primary sterol detected within the wild-type control. As expected, the *erg3Δ/Δ* strain lacked ergosterol, instead accumulating ergosta-7,22-dienol as the primary sterol species, which was not detectable in the wild type. The *P_ENO1_-*, *P_ACT1_-*, *P_YPT52_-*, and *P_VPS21_-ERG3* strains showed a progressive shift in sterol composition from the wild type to an *erg3Δ/Δ*-like profile, corresponding to the level of *ERG3* transcript produced. This confirmed that strains in the isogenic panel have a wide range of sterol Δ^5,6^-desaturase activity.

**TABLE 1:** Sterol profile of untreated Candida albicans strains

Sterol	*ERG3/ERG3*		*erg3Δ/Δ*		*Δ/P_VPS21_-ERG3*		*Δ/P_YPT52_-ERG3*		*Δ/P_ACT1_-ERG3*		*Δ/P_ENO1_-ERG3*	
	Mean(%)	SD	Mean(%)	SD	Mean(%)	SD	Mean(%)	SD	Mean(%)	SD	Mean(%)	SD
Ergosta-8,22,24(28)-trienol	0.2	0.0					0.1	0.0	0.1	0.1	0.1	0.0
Ergosta-5,8,22,24(28)-tetraenol	4.1	2.1					2.2	0.3	3.3	0.7	3.3	0.5
Ergosta-5,8,22-trienol	0.9	0.3					0.5	0.1	0.9	0.2	0.7	0.2
Zymosterol	1.9	0.2					0.5	0.0	2.5	0.8	2.4	0.9
Ergosta-8,22-dienol			3.7	0.2	1.7	0.1	2.4	0.3				
Ergosta-8,22,24(28)-trienol			3.1	0.5								
Ergosterol	79.2	3.3			17.3	0.9	45.0	2.4	75.8	3.3	85.0	1.4
Ergosta-7,22-dienol			69.5	3.0	58.0	2.4	34.1	1.4	3.5	0.4		
Ergosta-5,7,22,24(28)-tetraenol	0.7	0.1									0.5	0.1
Fecosterol (Ergosta-8,24(28)-dienol)	0.9	0.2	4.0	1.1	1.9	0.5	1.2	0.4	2.4	3.2	0.2	0.1
Ergosta-8-enol			1.1	0.2	0.6	0.0	0.3	0.0				
Ergosta-7,22,24(28)-trienol			0.9	0.1	0.4	0.2	0.2	0.0				
Ergosta-5,7,24(28)-trienol	0.9	0.4							0.5	0.5	1.0	0.1
Ergosta-5,7-dienol	2.7	0.1			0.7	0.0	1.0	0.1	1.5	0.1	1.4	0.1
Episterol (Ergosta-7,24(28)-dienol)	4.3	0.4	13.3	1.9	13.2	0.7	6.7	5.0	5.2	0.2	1.1	0.0
Ergosta-7-enol	0.2	0.0	3.7	0.1	4.6	3.3	2.4	0.2	0.4	0.1		
Lanosterol	3.5	0.5	0.4	0.1	1.3	0.1	2.7	0.2	3.1	0.1	3.5	0.2
4 methyl ergosta-8,24(28)-dienol	0.6	0.1	0.3	0.1	0.4	0.0	0.6	0.4	0.7	0.1	0.6	0.0
Total	100.0		100.0		100.0		100.0		100.0		100.0	
Total C-5-desaturated sterols	88.5		0.0		18.0		48.7		82.1		92.0	

**FIG 2:**
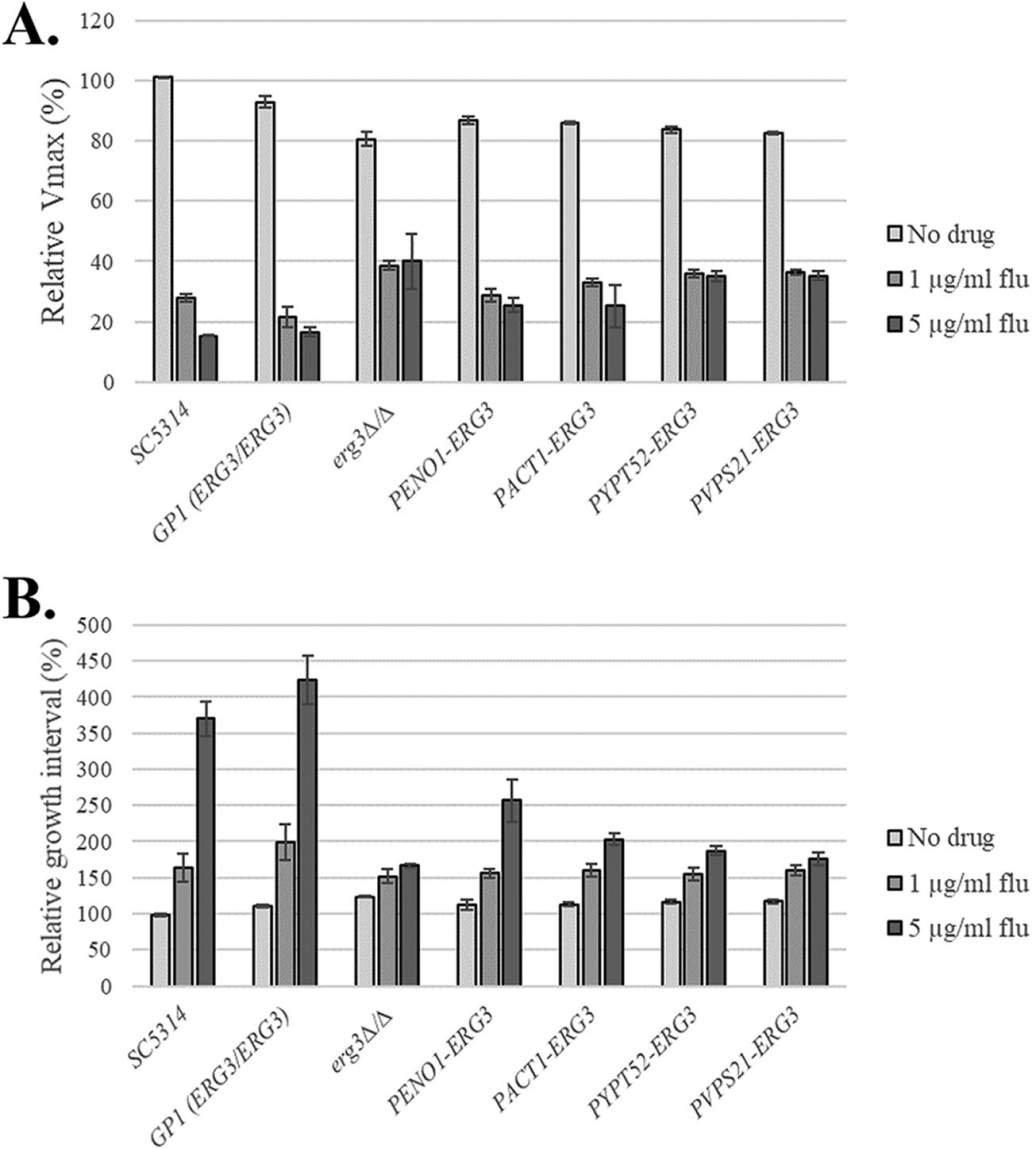
Candida albicans capacity to grow in the presence of fluconazole inversely correlates with *ERG3* transcript abundance. The indicated C. albicans strains were seeded into YPD medium at ∼1 × 10^5^ cells/mL supplemented with 1 or 5 μg/mL fluconazole or without drug (DMSO alone), dispensed into 96-well plates, and incubated at 30°C, and growth was monitored as OD_600_ at 30-min intervals. The wild-type strains SC5314 and GP1 as well as the *erg3*Δ/Δ mutant were included for reference. The maximum growth rate achieved after 8 h (*V*_max_) (A) and the time interval between reaching an OD of 0.25 and 0.75 (*T*_INT_) (B) were expressed as a percentage of the respective parameters of SC5314 in the absence of fluconazole. Data in all panels are the means and standard deviations from three biological replicates. The relative growth of each strain followed a similar pattern when the incubation temperature was changed to 37 or 41°C.

We next examined how *ERG3* transcript levels affect C. albicans fitness, stress tolerance, and morphogenesis. First, we compared the growth kinetics of the strains, primarily focusing on two parameters: (i) the maximum growth rate (*V*_max_) and (ii) the time elapsed between attaining an OD_600_ of 0.25 and 0.75 (*T*_INT_). In rich YPD (1% yeast extract, 2% peptone, 2% dextrose) medium at 30°C, the *V*_max_ of the *erg3Δ/Δ* mutant was approximately 80% of that of the wild-type reference strain (SC5314), with the promoter replacement strains ranging between 80 and 90% (Fig. 2A). *T*_INT_ was also slightly longer for the *erg3Δ/Δ* mutant (∼125% of that of SC5314), with the remaining strains including a second wild-type control (GP1) also having intermediate *T*_INT_ marginally longer than that of SC5314 (Fig. 2B). In contrast to the *erg3Δ/Δ* control strain (15, 21), all of the promoter replacement strains exhibited normal growth in the presence of excess calcium (Fig. 1B), indicating Erg3p sufficiency. However, on medium supplemented with SDS, the low *ERG3*-expressing strains (*P_VPS21_-ERG3* and *P_YPT52_-ERG3*) were sensitive, indicating insufficiency with respect to normal membrane integrity or function, while the *P_ACT1_-* and *P_ENO1_-ERG3* strains exhibited growth comparable to that of the wild type (Fig. 1B). As expected, the *erg3Δ/Δ* strain exhibited reduced hyphal growth compared to the wild type on both 10% fetal bovine serum (FBS) (Fig. 1C) and M199 (data not shown) agar at 37°C. Nonetheless, all of the promoter replacement strains formed normal or near-normal levels of hyphal growth on these media. The phenotypic profiles of each strain remained similar in relative terms with respect to hyphal growth, calcium, and SDS sensitivity when cultured at 30°C and at 35, 37, and 39°C (see Fig. S2 in the supplemental material), i.e., over a temperature range relevant to host colonization and infection. Thus, relatively low levels of *ERG3* transcription are sufficient to support C. albicans hyphal growth and at least a subset of the stress responses that are deficient in null mutants.

10.1128/mbio.00115-22.2 FIG S2 Low levels of *ERG3* transcription are sufficient to support Candida albicans stress tolerance at mammalian body temperature. C. albicans strains engineered to transcribe the *ERG3* ORF from either the *P_ENO1_*, *P_ACT1_*, *P_YPT52_*, or *P_VPS21_* promoter were suspended at 1 × 10^7^ cells/mL in sterile water and serially diluted 1:5, and each cell suspension was applied to YPD agar or YPD agar supplemented with 500 mM CaCl_2_, 0.05% SDS, or 5 μg/mL fluconazole. Plates were incubated at 30, 35, 37, or 39°C and imaged after 24 h or 48 h for the CaCl_2_ and SDS plates at 30°C. Download FIG S2, PDF file, 1.7 MB. Copyright © 2022 Regan et al. 2022 Regan et al. https://creativecommons.org/licenses/by/4.0/ This content is distributed under the terms of the Creative Commons Attribution 4.0 International license.

### Relatively minor reductions in Erg3p activity are sufficient to enhance Candida albicans growth in the presence of fluconazole.

**FIG 3:**
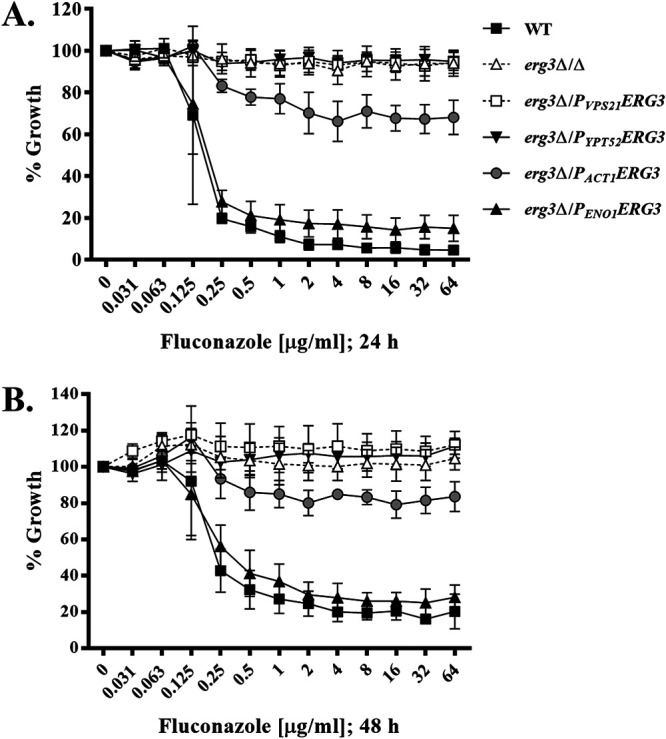
Erg3p activity correlates with Candida albicans sensitivity to fluconazole. The fluconazole susceptibility of the indicated C. albicans strains was compared using the CLSI broth microdilution protocol. The wild-type (WT) C. albicans strain SC5314 and *erg3*Δ/Δ mutant were included for reference. Growth was measured as the OD_600_ after 24 (A) or 48 (B) h of incubation at 35°C and expressed as a percentage of growth in the absence of drug (DMSO alone). Data in all panels are the means and standard deviations (error bars) from four biological replicates.

We next examined how titration of *ERG3* transcription affects C. albicans susceptibility to fluconazole using the standard CLSI broth microdilution protocol. Growth was quantified as optical density at 600 nm (OD_600_) after either 24 or 48 h of incubation. This revealed that the highest *ERG3*-transcribing strain, *Δ/P_ENO1_-ERG3*, exhibited sensitivity similar to that of the wild type at both the 24- and 48-h time points. Specifically, the MIC of fluconazole required to inhibit the growth of either strain, as well as the amount of residual growth at suprainhibitory concentrations of fluconazole (commonly known as trailing growth), were similar (Fig. 3A and B). Growth of the *Δ/P_ACT1_-ERG3* strain was only marginally reduced by even high concentrations of fluconazole at either the 24- or 48-h time point, whereas, similar to the *erg3Δ/Δ* strain, growth of the low-transcribing strains *Δ/P_VPS21_-ERG3* and *Δ/P_YPT52_-ERG3* was apparently unaffected by any concentration of fluconazole. These results demonstrate that relatively modest reductions in Erg3p activity are sufficient to substantially enhance C. albicans growth in the presence of fluconazole. In YPD broth, the addition of either 1 or 5 μg/mL fluconazole dramatically reduced the *V*_max_ of SC5314 to ∼27 and 15%, respectively, of that in drug-free medium (Fig. 2A). Fluconazole also had markedly reduced *V*_max_ for the *erg3Δ/Δ* mutant (by 50%) but to a much lesser extent than the wild-type controls. In the presence of fluconazole, *V*_max_ was intermediate for the promoter replacement strains and inversely proportional to the *ERG3* transcription level. For the wild-type strains, *T*_INT_ increased 3.5- to 4-fold in the presence of 5 μg/mL fluconazole (Fig. 2B). However, 5 μg/mL fluconazole extended *T*_INT_ of the *erg3Δ/Δ* mutant to just 1.5-fold longer than that for the wild type with no antifungal. In the presence of fluconazole, for all promoter replacements strains the *T*_INT_ was significantly shorter than that of the wild type, emphasizing their growth was less affected, with the lower expression strains being comparable to the *erg3Δ/Δ* mutant. The relative sensitivity of the strains was similar on YPD agar supplemented with fluconazole at temperatures between 30 and 39°C (Fig. S2), indicating the observed phenotypes are not temperature dependent.

Fluconazole exposure dramatically reduced the ergosterol content of the wild-type control, leading to the accumulation of several C-14–methylated intermediates, including lanosterol, eburicol, dimethyl zymosterol, and 14-methyl ergosta-8,24(28)-dien-3-6-diol (Table 2). However, as previously described, the *erg3Δ/Δ* strain did not produce the diol species in the presence of fluconazole. Furthermore, the *P_ENO1_-*, *P_ACT1_-*, *P_YPT52_-*, and *P_VPS21_-ERG3* strains all produced less diol than the wild type, at levels directly proportional to *ERG3* transcript abundance. Notably, the *Δ/P_ACT1_-ERG3* strain has no obvious deficiencies in stress tolerance or hyphal growth but could propagate much more effectively than the wild type in the presence of fluconazole. To a lesser extent this was also true of the *Δ/P_VPS21_-ERG3* and *Δ/P_YPT52_-ERG3* strains, which exhibited robust growth in the presence of fluconazole, with normal hyphal growth and calcium tolerance. However, these strains did exhibit sensitivity to membrane stress induced by SDS, indicating a mild but detectable fitness cost as a consequence of Erg3p insufficiency.

**TABLE 2:** Sterol profile of fluconazole-treated Candida albicans strains

Sterol	*ERG3/ERG3*		*erg3Δ/Δ*		*Δ/P_VPS21_-ERG3*		*Δ/P_YPT52_-ERG3*		*Δ/P_ACT1_-ERG3*		*Δ/P_ENO1_-ERG3*	
	Mean(%)	SD	Mean(%)	SD	Mean(%)	SD	Mean(%)	SD	Mean(%)	SD	Mean(%)	SD
Ergosta-5,8,22,24(28)-tetraenol	0.1	0.0			0.1	0.0	0.1	0.0	0.0	0.0	0.1	0.0
14 Methyl ergosta-tetraenol	0.7	0.1							0.2	0.0	0.3	0.0
Ergosta-8,22-dienol			0.2	0.1	0.1	0.0	0.0	0.0				
Ergosterol	0.9	0.1			0.8	0.0	1.0	0.1	1.4	0.3	0.8	0.4
Ergosta-8,22,24(28)-trienol			0.2	0.1								
14 Methyl ergosta-trienol	3.6	0.1	1.0	0.1	1.0	0.0	1.2	0.1	1.4	0.3	2.3	0.1
Ergosta-7,22-dienol			4.5	0.1	2.4	0.1	1.8	0.1				
Ergosta-5,7,22,24(28)-tetraenol	1.0	0.9							0.7	0.9	1.3	0.1
4,14 dimethyl zymosterol	18.3	0.3	45.9	0.4	44.5	0.3	43.8	0.8	42.2	0.4	37.3	0.4
14 Methyl fecosterol	5.8	0.4	3.3	0.1	3.8	0.3	4.1	0.2	4.6	0.0	4.8	0.1
4,4 dimethyl Ergosta 8,14,24(28)	1.1	0.0	0.7	0.0	0.7	0.0	0.8	0.0	0.8	0.0	0.9	0.2
14 Methyl ergosta-8,24(28)-dien-3-6-diol	14.3	1.7			0.3	0.0	1.0	0.1	2.7	0.1	4.2	0.2
Lanosterol	32.2	0.4	22.9	0.1	24.5	0.4	25.1	0.4	25.9	0.1	27.5	0.1
4,4,14 Tri methyl ergosta-trienol	0.8	0.1	0.7	0.0	0.7	0.1	0.7	0.1	0.8	0.0	0.8	0.1
Eburicol	21.2	0.4	20.6	0.1	21.0	0.8	20.5	0.6	19.2	0.3	19.9	0.2
Total	100.0		100.0		100.0		100.0		100.0		100.0	
Total C-5-desaturated sterols	17.0		0.0		1.2		2.0		5.1		6.6	

### Low levels of Erg3p activity are sufficient to support Candida albicans pathogenicity.

**FIG 4:**
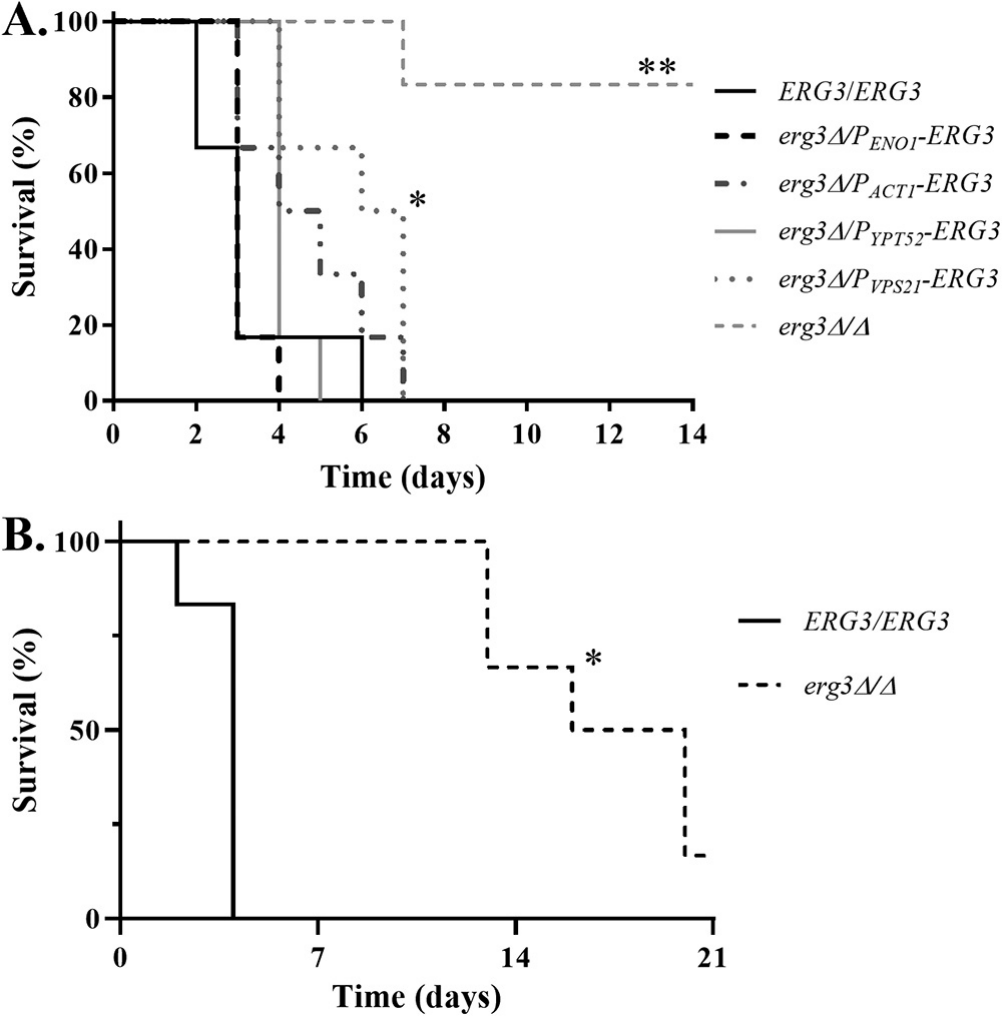
Low levels of *ERG3* expression are sufficient to support full virulence of Candida albicans. (A) Groups of 6 BALB/c mice were inoculated with ∼7 × 10^5^ CFU of each strain via lateral tail vein injection. The mice were then monitored three times daily for 14 days, and those showing distress were humanely euthanized. The survival of each group was then compared to that of SC5314 using the log rank test. *, *P = *0.0099; **, *P = *0.0006. (B) Groups of 6 CD1 mice were treated with 150 mg/kg cyclophosphamide at 3-day intervals and inoculated with ∼1 × 10^5^ CFU of the indicated strains via the tail vein 2 days after the first dose. Survival was compared as described above. *, *P = *0.0012.

We next compared the relative pathogenicity of the *P_ENO1_-*, *P_ACT1_-*, *P_YPT52_*-, and *P_VPS21_-ERG3* strains to wild-type and *erg3Δ/Δ* C. albicans in a mouse model of disseminated candidiasis. Groups of 6 BALB/c mice were infected with ∼7 × 10^5^ CFU of each strain via the lateral tail vein and monitored for 14 days. All mice infected with the wild-type strain succumbed to infection within 6 days and all those infected with the promoter replacement strains within 7 days (Fig. 4A), clearly establishing that even low levels of Erg3p expression are sufficient to support C. albicans virulence. In contrast, 5 of the 6 mice infected with the *erg3Δ/Δ* strain survived the duration of the experiment, confirming that complete loss of Erg3p activity impairs C. albicans virulence in this model. As previously reported (19), the *erg3Δ/Δ* strain could persist at high levels within the kidney tissue of the surviving mice (mean ± standard deviations [SD], 8.14 × 10^6^ ± 5.56 × 10^6^ CFU/g), even in the absence of overt signs and symptoms of disease. Nonetheless, the *erg3Δ/Δ* strain was able to cause lethality in leukopenic mice treated with cyclophosphamide, albeit with delayed onset compared to the wild type (Fig. 4B).

### Reduced Erg3p activity improves Candida albicans capacity to endure fluconazole exposure in immunosuppressed mice during disseminated infection.

**FIG 5:**
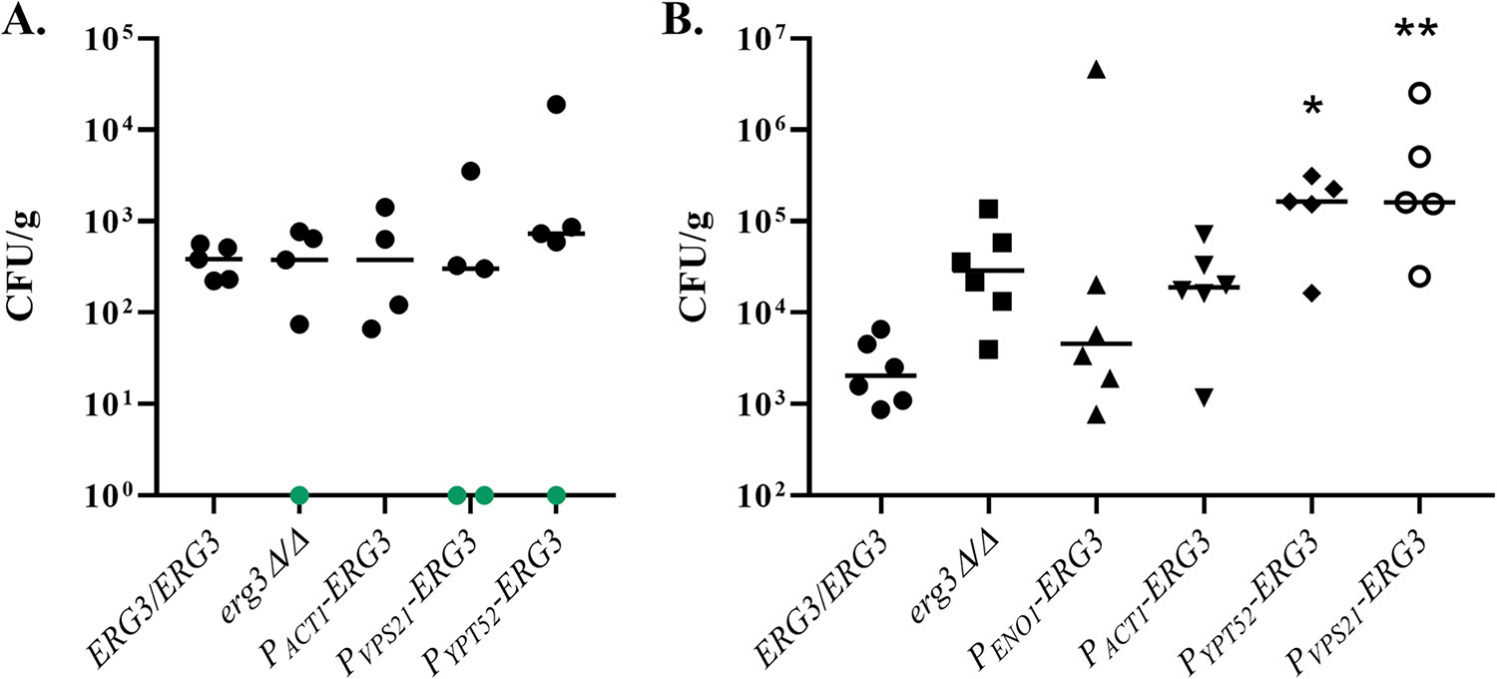
Low levels of *ERG3* expression reduce the antifungal efficacy of fluconazole against Candida albicans during disseminated infection of immunosuppressed mice. (A) Groups of 5 BALB/c mice were inoculated with ∼7 × 10^5^ CFU of the indicated strains via lateral tail vein injection, and fluconazole therapy was initiated 24 h later with 10 mg/kg/day on the first day followed by 5 mg/kg each day thereafter. Mice were euthanized on day 7 postinfection and levels of fungal colonization in the kidneys of each mouse quantified as number of CFU (normalized to organ weight). The median for each group is indicated by the crossbar. No significant differences were indicated between the groups in either experiment using the Kruskal-Wallis test. Note only 4 mice are plotted for the *P_ACT1_-ERG3* strain, as one mouse succumbed to infection before day 7 and is not plotted. (B) Groups of CD1 mice (*N* = 6) were treated with 150 mg/kg cyclophosphamide at 3-day intervals and inoculated with ∼1 × 10^5^ CFU of the indicated strains via the tail vein 2 days after the first dose. Fluconazole treatment and comparison of fungal colonization of kidney tissue was conducted as described above. Green points indicate numbers of CFU were below the level of detection. Note one mouse infected with *P_VPS21_-ERG3* and one with the *P_YPT52_-ERG3* strain succumbed to infection before day 7 and are not plotted. The median of all groups was compared to that of the *ERG3*/*ERG3* wild-type control using the Kruskal-Wallis multiple-comparison test (*P = *0.0028). For individual comparisons, *P = *0.0054 (*) and *P = *0.0018 (**).

**FIG 6:**
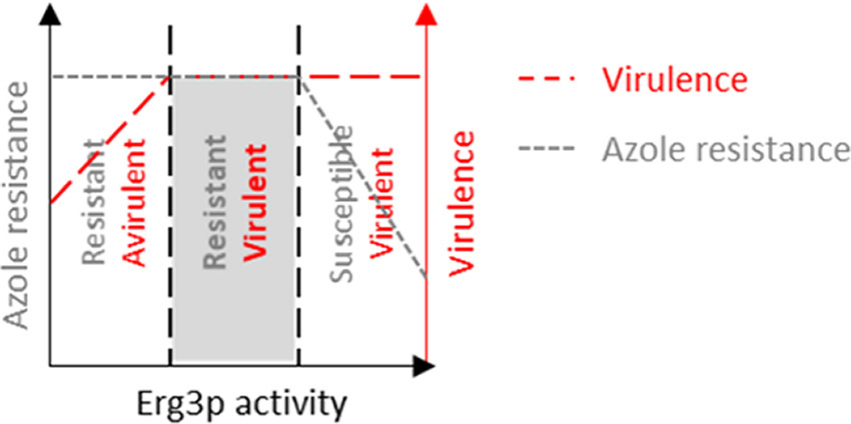
Proposed relationship between Erg3p activity, azole resistance, and C. albicans pathogenicity.

Finally, we determined how modulating Erg3p expression affects the capacity of C. albicans to survive fluconazole exposure within mammalian tissue, using the standard mouse model of disseminated infection. In the first experiment, groups of 5 mice were infected with either the wild-type, *P_ENO1_-ERG3*, *P_ACT1_-ERG3*, *P_YPT52_*-*ERG3*, *P_VPS21_-ERG3*, or *erg3Δ/Δ* strain as described above, and fluconazole treatment was initiated 24 h later at 10 mg/kg of body weight on day 1, followed by 5 mg/kg on each subsequent day. Only one mouse (infected with the *P_ACT1_-ERG3* strain) succumbed to infection during the course of this experiment and was found to have very high fungal burden within the kidney tissue. Of those surviving the duration (to day 7 postinfection), there was no significant difference between the wild-type, *erg3Δ/Δ*, and promoter replacement strains in the overall levels of kidney tissue colonization (Fig. 5A). In a second experiment, a lower dose of fluconazole (3.5 mg/kg/day) was used to treat groups of 5 mice infected with each strain. Again, just one mouse, this time infected with the *P_VPS21_-ERG3* strain, succumbed within the 8-day experiment. Of the surviving mice, those infected with the *P_VPS21_-ERG3* and *P_YPT52_-ERG3* strains were the most heavily colonized (Fig. S1); however, the differences were small and not statistically significant compared to the wild-type control. Thus, low levels of Erg3p expression do not appear to confer a profound survival advantage upon the fungus in this model. Finally, we conducted a similar experiment using immunosuppressed mice (*N* = 6), rendered leukopenic through cyclophosphamide treatment. Fluconazole treatment (10 mg/kg on first day, followed by 5 mg/kg/day) was initiated 24 h postinfection, and surviving mice were euthanized after 6 days of treatment. All mice infected with the wild-type control had detectable levels of fungal colonization within their kidneys. Two mice, one infected with the *P_YPT52_-ERG3* and one with the *P_VPS21_-ERG3* strain, succumbed to infection during the 6-day treatment period. All other mice infected with the promoter replacement strains had higher levels of tissue colonization than the wild type, with number of CFU/gram inversely correlated with *ERG3* transcript abundance and Erg3p activity detected in the sterol profiles (Fig. 5B). Kidney colonization by the *P_VPS21_-ERG3* strain was approximately 100-fold higher than that of the wild type, clearly establishing that low levels of Erg3p activity confer a substantial advantage upon C. albicans with respect to surviving fluconazole therapy within the immunosuppressed host. Notably, the *erg3Δ/Δ* mutant also had higher levels of colonization within the kidneys than the wild-type control but were lower than those for the promoter replacement strains. In addition, null mutant-infected mice lost less weight and exhibited less severe symptoms of disease than the other strains. These results indicate that reduction of Erg3p activity to levels below that of strain SC5314 confers a greater advantage than complete loss of function not only with respect to virulence but also to C. albicans capacity to endure azole therapy (Fig. 6).

10.1128/mbio.00115-22.1 FIG S1 Low levels of *ERG3* expression does not confer a substantial fluconazole resistance upon Candida albicans during disseminated infection of immunocompetent mice. Groups of 4 or 5 BALB/c mice were inoculated with ∼7 × 10^5^ CFU of each strain via lateral tail vein injection and fluconazole therapy initiated 24 h later with 3.5 mg/kg/day. After 7 days postinfection, the mice were euthanized and levels of fungal colonization in each mouse quantified as colony-forming units (normalized to weight). The median for each group is indicated by the crossbar. No significant differences were indicated between the groups in either experiment using the Kruskal-Wallis test. Red points indicate an animal that succumbed to infection before the end of the experiment; green points indicate CFUs were below the level of detection. Download FIG S1, PDF file, 0.1 MB. Copyright © 2022 Regan et al. 2022 Regan et al. https://creativecommons.org/licenses/by/4.0/ This content is distributed under the terms of the Creative Commons Attribution 4.0 International license.

## DISCUSSION

While complete loss of Erg3p activity has been shown to restore the capacity of C. albicans to grow in the presence of the azole antifungals, the resulting changes in membrane sterol composition also cause some physiological dysfunction. We previously reported that complete loss of Erg3p activity is not necessary to confer azole tolerance *in vitro* (19, 22, 23). The results of this study have further established that the capacity of C. albicans to grow in the presence of the azole antifungals is inversely correlated with Erg3p activity. In fact, relatively small reductions in Erg3p activity are sufficient to substantially enhance C. albicans growth in the presence of the azole antifungals. In contrast, a subset of the other phenotypes that have been associated with *ERG3* insufficiency, specifically hypersensitivity to calcium and hyphal growth defects, require more dramatic reductions in Erg3p activity, while sensitivity to the membrane-disrupting agent SDS is observed with moderate reductions in *ERG3* transcription. Thus, different thresholds of Erg3p activity are associated with each phenotype. This is potentially important, as it implies modulation of Erg3p expression or activity reduces C. albicans sensitivity to the azole antifungals without compromising pathogenic fitness. Furthermore, we have observed significant variation in basal as well as azole-inducible levels of *ERG3* transcript abundance among a collection of clinical isolates compared to the SC5314 reference strain (G. Palmer, unpublished results), although the extent to which variation in *ERG3* transcript abundance influences the ability of individual isolates to tolerate the azole antifungals remains to be established.

Perhaps muddying the waters further is our recent finding that the enhanced growth of Erg3p-deficient C. albicans strains in the presence of the azole antifungals has some characteristics of the trailing growth phenomenon and some of true azole resistance (22). Trailing growth is the residual growth that can often be observed at supra-MICs of the azoles and is insensitive to further increases in antifungal concentration. With the standard CLSI susceptibility testing method, trailing isolates usually appear azole susceptible at the 24-h time point, but continued growth makes them appear resistant at 48 h (24). Careful inspection of the fluconazole dose-response curve revealed that the inflection point of the dose versus percent growth plot for the C. albicans strains expressing moderate levels of *ERG3* was in the same range as that for the wild type; however, the amount of growth observed at antifungal concentrations above the inflection point increased as *ERG3* transcript abundance decreased. Furthermore, the amount of growth observed at fluconazole concentrations above the inflection point was not dose dependent. Analysis of growth kinetics also revealed that the growth rate of the *erg3Δ/Δ* mutant was reduced in the presence of fluconazole but to a much lesser extent than that of the wild type. These data support categorizing the continued growth of *erg3*-deficient strains in the presence of ordinarily inhibitory concentrations of the azoles as a form of trailing growth. While patients and experimental animals infected with trailing isolates generally respond well to treatment with the azoles (25, 26), two recent reports indicate that trailing isolates are less readily cleared from infected tissues following azole therapy than nontrailing isolates (27, 28). Either way, this definition seems unsatisfactory with the standard CLSI protocol, as the *erg3Δ/Δ* mutant and strains with low levels of Erg3p activity exhibit no obvious reduction in fungal growth at any concentration of fluconazole, even at the 24-h time point, when trailing growth is usually absent.

Previous studies have suggested that loss of Erg3p function is associated with reduced C. albicans virulence in the standard (immunocompetent) mouse model of disseminated infection (9, 13, 16, 19). The avirulence of *erg2Δ/Δ*, *erg24Δ/Δ*, and *erg3Δ/Δ* mutants in the standard mouse model of disseminated C. albicans infection (19, 29) suggests that while ergosterol is dispensable for *in vitro* growth, it is required to cause invasive disease within the mammalian host. Here, we demonstrated that our *erg3Δ/Δ* strain can cause lethal disseminated infection in immunosuppressed mice, but the delayed onset is consistent with diminished virulence rather than avirulence. Thus, there is no absolute requirement for Erg3p function, which explains the isolation of *erg3*-deficient mutants from patients with azole-refractory C. albicans infections (2, 9–11). Thus, the first surprising result from our *in vivo* studies is that even very low levels of *ERG3* expression apparently are sufficient to support full or near-full virulence of C. albicans despite substantial reductions in ergosterol content and the accumulation of dienol intermediates such as ergosta-7,22-dienol. This implies that C. albicans is highly tolerant of perturbations to the sterol biosynthetic pathway, with even low levels of ergosterol sufficient to support pathogenicity in the mouse model of disseminated infection.

Our data also suggest that the C-14–methylated sterols that accumulate upon S14DM inhibition can support C. albicans growth *in vitro*, albeit at a reduced rate, but that high levels of sterol diol accumulation (associated with high levels of Erg3p activity) are extremely detrimental to growth. In contrast, we found that azole-mediated inhibition of S14DM is similarly efficacious on both the high and low Erg3p-expressing strains during disseminated infection of otherwise healthy mice. Taken alone this implies that the C-14–methylated sterols that accumulate are not sufficient to support fungal proliferation or virulence during disseminated infection of the mammalian host, irrespective of the Erg3p-mediated production of diols. However, reduced Erg3p activity confers a clear survival advantage to C. albicans during azole therapy in the immunosuppressed host. This implies that the C-14–methylated sterols that accumulate upon azole exposure are sufficient to support C. albicans proliferation in the absence of a robust, sustained immune response. While the studies described here were conducted using a mouse model of disseminated infection, it is also important to consider the site of C. albicans infection, as the physical and immunological stresses encountered within different tissue microenvironments are quite distinct. These factors are, in turn, likely to provide a context that affects the capacity of C. albicans to proliferate and cause disease in the absence of ergosterol or with abnormal or suboptimal sterol profiles. This would include the dienols accumulated by *erg3*-deficient strains, the C-14–methylated sterols formed upon S14DM inhibition, and possibly the level of injury caused by the accumulated sterol-diol. Notably the primary ecological niche of C. albicans is the reproductive and gastrointestinal tracts, body sites at which activation of the immune system is often constrained and/or subject to a delicate and complex set of controls to prevent immunopathogenesis. These mucosal environments are unlikely to impose such intense stress upon the fungus as encountered following entry into and dissemination via the bloodstream and may therefore be permissive for the selection of Erg3p-deficient strains. Recently, we reported that despite significant virulence defects in the standard mouse model of disseminated infection, our *erg3Δ/Δ* mutant can colonize to the same extent as the *ERG3^+^* controls in a mouse model of vaginal candidiasis and is apparently resistant to azole treatment (19). These data are consistent with niche-specific requirements for Erg3p function with respect to pathogenicity. They also further support the notion that the *erg3Δ/Δ* mutants’ capacity to endure the azole antifungals is context dependent. Finally, the studies described here were conducted using the SC5314 reference strain background. It is likely that the capacity of C. albicans (and other infectious fungi) to remain viable following depletion of ergosterol, utilize accumulated biosynthetic intermediates, and tolerate the presence of abnormal and/or dysfunctional sterol species is subject to a variety of genetic and epigenetic modifiers. This could include strain-specific differences in the type and intensity of the stress responses activated upon membrane perturbation or in the expression/catalytic activity of individual enzymes within the sterol biosynthetic pathway (other than Erg3p and Erg11p) that influence sterol composition. Sanglard and colleagues reported an *erg3* null mutant that was apparently fully virulent and azole resistant during disseminated infection of immunocompetent mice (10), indicating loss of Erg3p function was well tolerated in this strain background. As such, there are likely a number of fungal and host-specific factors that influence the requirement for Δ^5,6^-desaturase function during infection of the mammalian host. The identification of genetic and physiological modifiers of the *erg3*-related phenotypes remains a key goal.

## MATERIALS AND METHODS

### Growth conditions.

C. albicans was routinely grown on YPD medium (1% yeast extract, 2% peptone, 2% dextrose) at 30°C, supplemented with uridine (50 μg/mL) when necessary. Transformant selection was carried out on minimal YNB medium (6.75 g/liter yeast nitrogen base without amino acids, 2% dextrose, 2% Bacto agar), supplemented with the appropriate auxotrophic requirements as described for S. cerevisiae (30) or 50 μg/mL uridine.

### Plasmid construction.

Plasmid pLUX (31) was kindly provided by William Fonzi (Georgetown University). Plasmid pKE1 (32) was previously described. All oligonucleotides used in this study are listed in Table S1 in the supplemental material.

### Candida albicans strains.

The *ERG3*/*erg3Δ:ARG4* and *erg3Δ*/*Δ* strains were constructed in a previous study (19). Transformation of C. albicans with DNA constructs was performed using the lithium acetate method (33). *ERG3* promoter replacement cassettes were amplified from p*HIS1-P_ENO1_*, p*HIS1-P_TEF1_*, *pHIS1-P_ACT1_*, p*HIS1-P_VPS21_*, p*HIS1-P_ENO1_*, and p*HIS1-P_YPT52_* (20), using the ERG3PRF and ERG3PRR primer pair to incorporate homologous flanking sequences. The promoter of the remaining *ERG3* allele in the *ERG3*/*erg3Δ:ARG4* heterozygous strain was then replaced with each promoter replacement cassette to produce the *erg3Δ:ARG4*/*HIS1:P_x_-ERG3* strains. Replacement of the native promoter was indicated by the absence of a 2,199-bp product using primer pair ERG3AMPF and ERG3DETR. Correct integration was then further confirmed by PCR using the ERG3DETR primer with either ENO1prSEQF, TEF1prSEQF, ACT1prSEQF, VPS21prSEQF, or YPT52prSEQF. All promoter replacement strains were then made prototrophic by integrating NheI linearized pLUX (31), and individual transformant clones were isolated through selection on medium lacking uracil or uridine. Correct integration of pLUX (and, thus, full restoration of the *URA3-IRO1* locus) was confirmed by the amplification of a 2.1-kb product following PCR amplification of purified genomic DNA with the LUXINTDETF and LUXINTDETR primer pair.

### Stress resistance and hyphal growth assays.

For stress resistance analysis, C. albicans was grown overnight in YPD at 30°C. Cells were washed in sterile deionized water, cell density was adjusted to 10^7^ cells/mL, and 1:5 serial dilutions were performed in a 96-well plate. Cells were then applied to agar plates using a sterile multipronged applicator. Resistance to different stresses was determined on YPD agar containing 5 μg/mL fluconazole, 0.05% SDS, or 500 mM CaCl_2_, and plates were incubated at 30°C for 48 h before imaging. For hyphal growth analysis, 2.5 μL from a 10^7^-cells/mL cell suspension was spotted on 10% FBS (fetal bovine serum) or M199 agar plates and then incubated for 96 h at 37°C before imaging.

### RNA extraction.

Each C. albicans strain was grown overnight in YPD at 30°C, subcultured to an OD_600_ of 0.2, and incubated at 30°C with shaking for 6 h. Cells were collected by centrifugation at 3,500 rpm for 5 min. The supernatant was poured off and cells pellets were stored at −80°C. RNA was extracted from the cell pellets using the hot phenol method described by Schmitt et al. (34). RNA pellets were eluted in nuclease-free water, and quantity and purity were determined spectrophotometrically by measuring absorbance at 260 nm and 280 nm.

### qRT-PCR.

cDNA was synthesized using the Verso cDNA synthesis kit (Thermo Scientific) with random hexamers according to the manufacturer’s instructions. Quantitative PCR was performed with primer pairs that bind within the *ACT1* (ACT1-FWD-qRT-PCR + ACT1-RVS-qRT-PCR) coding sequences using the Maxima SYBR green/ROX qPCR master mix (2×) (Thermo Scientific) as indicated by the manufacturer. Reactions were completed in a CFX96 real-time system (Bio-Rad). Expression levels of *CDR1*, *MDR1*, and *ERG11* among the strains were compared to those of *ACT1* (normalizing gene) using the 2^−ΔΔ^*^CT^* method (35).

### Antifungal susceptibility testing.

Antifungal susceptibility testing of all the strains included in this study was performed using the broth microdilution method described in CLSI document M27-A3 (36) in a 96-well plate format. All drugs for susceptibility testing used in this study were diluted in dimethyl sulfoxide (DMSO) in 2-fold dilutions at 200 times the final concentration. RPMI 1640 medium (Sigma-Aldrich) was prepared according to the CLSI document (36); the medium was buffered with morpholinepropanesulfonic acid (MOPS) and pH adjusted using NaOH and HCl. Plates were incubated without shaking for 24 or 48 h at 25°C or 35°C. The content of each well was carefully resuspended by pipetting up and down before the OD_600_ was measured using a BioTek Cytation 5 plate reader.

### Sterol extraction and quantitation.

Strains were grown overnight at 37°C and 200 rpm for 16 h and then subcultured to an OD_600_ of 0.25 into 10 mL YPD broth supplemented with 5 mg/liter fluconazole or 0.5% DMSO vehicle alone and then grown for 6 h at 37°C. Nonsaponifiable lipids were extracted using alcoholic KOH as reported previously (37). Samples were dried in a vacuum centrifuge (Heto) and were derivatized by the addition of 100 μL 90% N,O-Bistrifluoroacetamide–10% tetramethylsilane (TMS) (Sigma), 200 μL anhydrous pyridine (Sigma) and heating for 2 h at 80°C. TMS-derivatized sterols were analyzed and identified using GC-MS (Thermo 1300 GC coupled to a Thermo ISQ mass spectrometer; Thermo Scientific) with reference to retention times and fragmentation spectra for known standards. GC-MS data files were analyzed using Xcalibur software (Thermo Scientific) to determine sterol profiles for all isolates and for integrated peak areas. Percentages of total sterols are given as the mean from 3 replicates.

### Growth kinetic analysis.

Each strain was subcultured into YPD broth supplemented with 1 or 5 μM fluconazole or 0.5% DMSO vehicle at approximately 1 × 10^4^ cells/mL, and 200 μL of each cell suspension was transferred to the wells of a round-bottom 96-well plate. The plate was then incubated at 30°C within a BioTek Cytation 5 plate reader and OD_600_ read at 30-min intervals. Background was measured from a well with medium alone (no cells) and subtracted from each reading before the OD_600_ was plotted as a function of time. The post-8-h *V*_max_ (i.e., *V*_max_ following the initiation of azole-mediated growth inhibition) and *T*_INT_ (period of time elapsed between reaching an OD of 0.25 and 0.75) was calculated using the Gen 5 reader software and expressed as a percentage of the same parameters for the SC5314 wild-type control strain grown in the absence of fluconazole. Each experiment was conducted on three separate occasions, and the means and standard deviations of *V*_max_ and *T*_INT_ are presented.

### Ethics statement.

The animals used in this study were housed in Association for Assessment and Accreditation of Laboratory Animal Care (AAALAC)-approved facilities at the University of Tennessee Health Science Center (UTHSC). The Institutional Animal Care and Use Committee (IACUC) at UTHSC approved use of all animals and procedures (IACUC protocol number 19–0115). Mice were given standard rodent chow and water *ad libitum*. Mice were monitored daily for signs of distress, including noticeable weight loss, lethargy, and body condition score. The IACUC at UTHSC uses the Public Health Service (PHS) *Policy on Humane Care and Use of Laboratory Animals* (38) and the *Guide for the Care and Use of Laboratory Animals* (39) as a basis for establishing and maintaining an institutional program for activities involving animals. To ensure high standards for animal welfare, the IACUC at UTHSC remains compliant with all applicable provisions of the Animal Welfare Act (AWAR), guidance from the Office of Laboratory Animal Welfare (OLAW), and the American Veterinary Medical Association Guidelines on Euthanasia.

### Mouse model of disseminated candidiasis.

C. albicans strains were grown overnight in YPD cultures at 30°C with shaking, washed twice, and resuspended in sterile endotoxin-free phosphate-buffered saline (PBS). BALB/c or CD1 mice were inoculated via lateral tail vein injection with ∼7 × 10^5^ cells in 100 μL of cell suspension. Viable cell counts of each inoculum were confirmed by plating appropriate dilutions onto YPD agar plates and counting the colonies formed after 48 h. In some experiments mice were rendered leukopenic by administering 150 mg/kg cyclophosphamide 2 days before infection and every third day thereafter via intraperitoneal injection. For cyclophosphamide-treated mice the infectious dose was reduced to 1 × 10^5^ *Candida* cells. Groups of six mice were infected with each fungal strain as described above and monitored for 14 or 21 days postinfection, and those showing signs of distress were humanely sacrificed. Animals surviving to the end of the experiment were euthanized and kidneys extracted, weighed, and homogenized in PBS. Serial dilutions of kidney homogenate were plated on YPD agar plates containing 50 μg/mL chloramphenicol. The number of CFU/gram of kidney tissue was determined from the number of colonies on the plates after 48 h. For experiments involving treatment with fluconazole, 24 h postinfection, mice were treated with 10 mg/kg fluconazole in sterile PBS, followed by 5 mg/kg on each subsequent day, or with vehicle alone (PBS plus 1% DMSO) via intraperitoneal injection until day 7 postinfection. Mice were monitored daily, and those showing distress were euthanized. On day 8 postinfection, all surviving animals were sacrificed, their kidneys removed, and fungal burdens determined as described previously. In a second experiment mice were treated with 3.5 mg/kg/day fluconazole.
