# Supplementary material for: Titration of C-5 Sterol Desaturase Activity Reveals Its Relationship to Candida albicans Virulence and Antifungal Susceptibility Is Dependent upon Host Immune Status
Source: mBio. 2022 Apr 5;13(2):e00115-22. doi: 10.1128/mbio.00115-22 (PMC9040724; doi:10.1128/mbio.00115-22)
Supplement: FIG S2 [file mbio.00115-22-sf002.pdf]

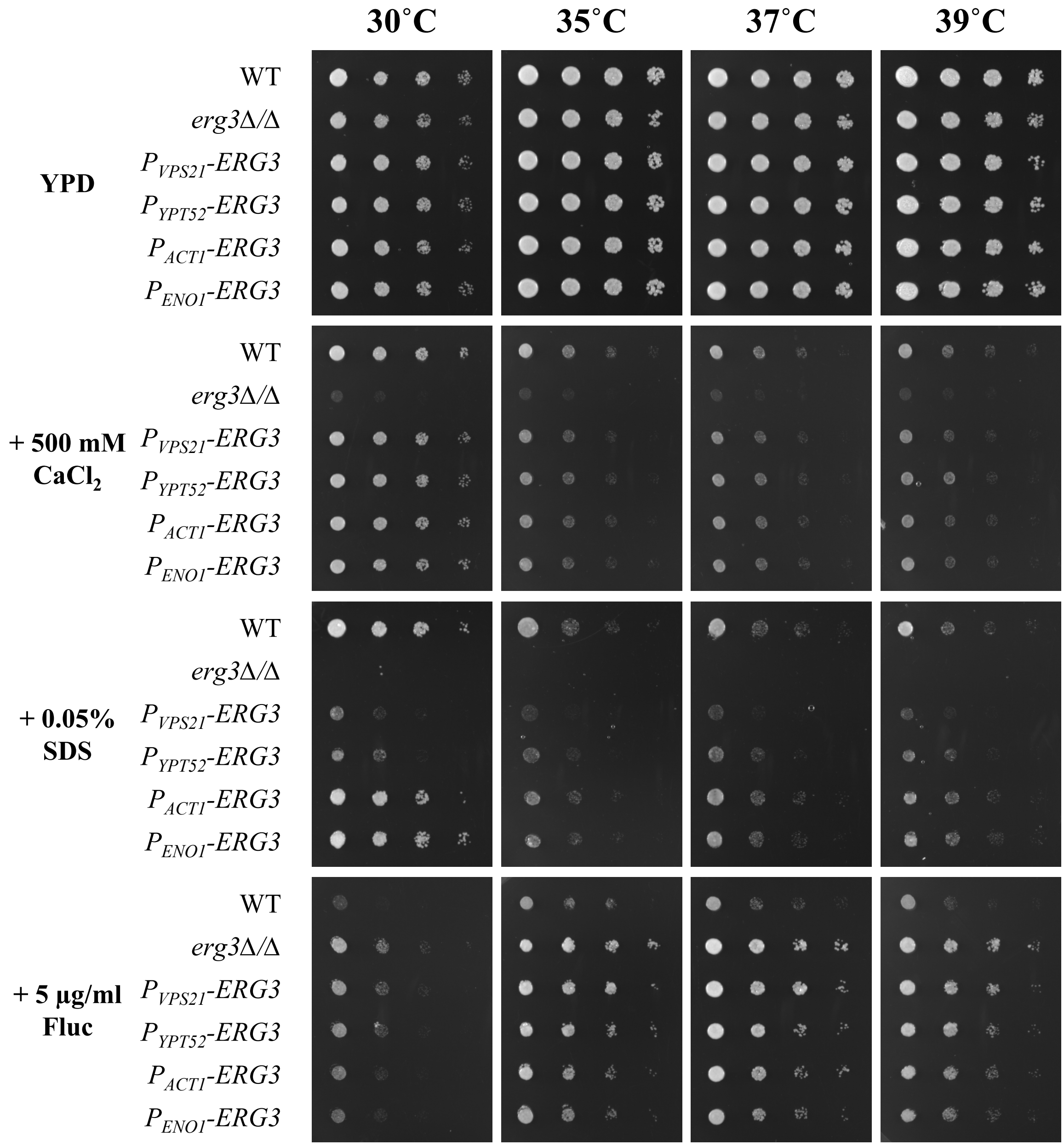


**Figure S2. Low levels of *ERG3* transcription are sufficient to support *Candida albicans* stress tolerance at mammalian body temperature.** *C. albicans* strains engineered to transcribe the *ERG3* ORF from either the $P_{ENO1}$, $P_{ACT1}$, $P_{YPT52}$ or $P_{VPS21}$ promoters were suspended at 1 X $10^7$ cells/ml in sterile water, serially diluted 1:5, and each cell suspension applied to YPD agar, or YPD agar supplemented with 500 mM $CaCl_2$, 0.05% SDS or 5 µg/ml fluconazole. Plates were incubated at 30, 35, 37 or 39°C and imaged after 24 hours, or 48 hours for the $CaCl_2$ and SDS plates at 30°C.
