# Supplementary material for: Titration of C-5 Sterol Desaturase Activity Reveals Its Relationship to Candida albicans Virulence and Antifungal Susceptibility Is Dependent upon Host Immune Status
Source: mBio. 2022 Apr 5;13(2):e00115-22. doi: 10.1128/mbio.00115-22 (PMC9040724; doi:10.1128/mbio.00115-22)
Supplement: FIG S1 [file mbio.00115-22-sf001.pdf]

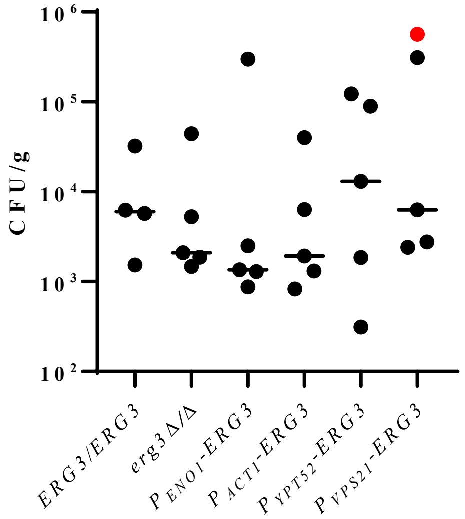

Figure S1. Low levels of *ERG3* expression does not confer a substantial fluconazole resistance upon *Candida albicans* during disseminated infection of immunocompetent mice. Groups of 4 or 5 BALB/c mice were inoculated with ~7 X 10^5 CFU of each strain via lateral tail vein injection and fluconazole therapy initiated 24 hours later with 3.5 mg/Kg/day. After 7 days post-infection, the mice were euthanized and levels of fungal colonization in each mouse quantified as colony forming units (normalized to weight). The median for each group is indicated as by the cross bar. No significant differences were indicated between the groups in either experiment using the Kruskal-Wallis test. Red points indicate an animal that succumbed to infection before the end of the experiment; Green points indicate CFU's were below the level of detection.
